# Factors associated with low birthweight: a case-control study in a city of Minas Gerais

**DOI:** 10.11606/s1518-8787.2020054002065

**Published:** 2020-07-15

**Authors:** Érica Cesário Defilipo, Paula Silva de Carvalho Chagas, Andreia Peraro-Nascimento, Luiz Cláudio Ribeiro

**Affiliations:** I Universidade Federal de Juiz de Fora campus Governador Valadares Instituto de Ciências da Vida Governador ValadaresMG Brasil Universidade Federal de Juiz de Fora campus Governador Valadares. Instituto de Ciências da Vida. Departamento de Fisioterapia. Governador Valadares, MG, Brasil; II Universidade Federal de Juiz de Fora Faculdade de Fisioterapia Programa de Pós-graduação em Ciências da Reabilitação e Desempenho Físico-funcional Juiz de ForaMG Brasil Universidade Federal de Juiz de Fora. Faculdade de Fisioterapia. Programa de Pós-graduação em Ciências da Reabilitação e Desempenho Físico-funcional. Juiz de Fora, MG, Brasil; III Universidade Federal de Juiz de Fora campus Governador Valadares Instituto de Ciências da Vida Governador ValadaresMG Brasil Universidade Federal de Juiz de Fora campus Governador Valadares. Instituto de Ciências da Vida. Departamento de Farmácia. Governador Valadares, MG, Brasil; IV Universidade Federal de Juiz de Fora Departamento de Estatística Programa de Pós-Graduação em Saúde Coletiva Juiz de ForaMG Brasil Universidade Federal de Juiz de Fora. Departamento de Estatística. Programa de Pós-Graduação em Saúde Coletiva. Juiz de Fora, MG, Brasil

**Keywords:** Low Birth Weight Infant, Risk Factors, Environmental Exposure, Socioeconomic Factors, Case Studies and Controls, Maternal and Child Health

## Abstract

**OBJECTIVE:**

To analyze the many factors regarding socioeconomic and healthcare-related variables linked to maternal diseases and the possible impact of the environmental disaster of Mariana, given the prenatal exposure to different water sources for human consumption that were associated with low birthweight in full-term live births in the Municipal Hospital of Governador Valadares, Minas Gerais.

**METHODS:**

Case-control study, carried out with live births at the Municipal Hospital of Governador Valadares, from May 2017 to July 2018. The case group consisted of full-term live births and low birthweight, and the control group consisted of full-term live births with adequate weight, matched by gender and date of birth. For each case, two controls were selected. Data collection was performed through interviews with the puerperal women, and complementary information was obtained by analyzing the prenatal card and medical records. For data analysis, logistic regression was performed.

**RESULTS:**

The study included 65 live births from the case group and 130 from the control group. After the analysis was adjusted for other factors under study, we found that the higher risks of low birthweight are associated with the first childbirth (OR = 2.033; 95%CI = 1.047–3.948; p = 0.036), smoking during pregnancy (OR = 2.850; 95%CI = 1.013–8.021; p = 0.047) and consumption of water supplied by the municipalities affected by the tailings from the Fundão dam failure (RC = 2.444; 95%CI = 1.203–4.965; p = 0.013).

**CONCLUSIONS:**

The variables “water consumed during pregnancy,” “previous pregnancies” and “maternal smoking” were associated with low birthweight in the population studied. The importance of epidemiological studies that assess water quality and its adverse health effects is reinforced, as well as greater prenatal control of first-time pregnant women and greater support of policies against smoking, especially during pregnancy.

## INTRODUCTION

Each year more than twenty million children with low birthweight are born worldwide, which is defined as weighing less than 2.5 kg^1 , 2^ . Low birthweight is considered an important risk marker for infant mortality, besides contributing to morbidities such as infectious diseases, cognitive and neurological dysfunctions, developmental delay and greater probability of developing chronic diseases^3^ .

Low birthweight rates in Brazil, considering the analysis of the period from 1996 to 2011, show stability of about 8%. When analyzing regionality, highest rates were found in the most developed regions, Southern and Southeastern, and a significant increase was observed in the less developed regions, North, Northeast and Midwest^4^ . The reasons for the increase in these rates, considering the improvement of social and maternal and child health indicators in recent years, are not well-known^5^ .

Low birthweight is considered a complex public health problem due to its multifactorial etiology. A review study on low birthweight in the Americas showed that most studies published recently agree with the association of sociodemographic, biological and behavioral factors. Studies that refer to the association of low birthweight with environmental risk factors, such as exposure to air, water or soil pollution, are gaining importance^1^ .

The association of low birthweight with air pollution has been reported in the literature^1 , 6^ , but few studies have evaluated the relationship of this outcome with water quality. In view of the socioenvironmental tragedies that occurred in Minas Gerais, such as the Fundão dam failure in Mariana, research is needed to assess water quality and its adverse health effects. At some stages of life, the damage caused to health by pollution can be irreversible. This situation becomes more serious when we refer to maternal and child health, due to the interference in reproduction and gestational and living conditions of the infant^7^ .

This study aimed to analyze the many factors regarding socioeconomic and healthcare-related variables linked to maternal diseases and the possible impact of the environmental disaster that took place in Mariana, given the prenatal exposure to different water sources for human consumption that were associated with low birthweight in full-term live births in the Municipal Hospital of Governador Valadares, Minas Gerais.

## METHODS

Case-control study carried out with live births at the Municipal Hospital of Governador Valadares, from May 2017 to July 2018. This hospital was chosen as it is linked to the Brazilian Unified Health System and it is the only one with a neonatal intensive care unit in the region, considered a reference for the cities of the Rio Doce Valley. Cases were considered live births at term, with gestational age equal to or greater than 37 weeks and less than 42 weeks and weighing less than 2,500 grams (low birthweight), while the controls were full-term live births weighing 2,500 grams or more (adequate birthweight), matched by gender and date of birth. For each case, two controls were selected. Live births with congenital malformations, genetic syndromes, progressive diseases and lesions of the nervous system, diagnosed or suspected at birth, were excluded from the study. When there were more than two newborns who met the inclusion criteria, choosing was random. When there were no two controls for pairing, the case was not included in the study.

Data collection was performed through structured interviews with the puerperal women, still during hospitalization, within 24 to 48 hours after delivery. Complementary information was obtained by analyzing the prenatal card and the records of the puerperal mother and newborn. Data were collected by previously trained researchers.

Low birthweight was considered as a dependent variable. The independent variables were organized into five blocks, according to the explanatory model presented in Figure , which contains the categorization form of each variable studied. To analyze the variable “water consumed during pregnancy,” the fact that the participants lived in different municipalities was considered; while some were affected by the tailings from the Fundão dam failure, others were not. For this reason, this variable was categorized into 1) mineral water, supplied from mines, wells or cisterns or from the water supply service of municipalities not affected by mud; and 2) water from the supply service of the affected municipalities. The categorization of the variables “number of prenatal appointments” and “first prenatal appointment” was defined based on the one proposed by the Ministry of Health, which determines the beginning of prenatal care up to the sixteenth week of gestation and a minimum of six appointments^8^ . The variable “number of appointments” was categorized according to what would be recommended (six appointments or more) or not recommended (less than six appointments) and the variable “first prenatal appointment” was categorized as up to 16 weeks and more than 16 weeks^9^ ( Figure ). Alcohol dependence was detected using the CAGE questionnaire, considering as dependent women who presented at least one affirmative answer^10^ .

**Figure:**
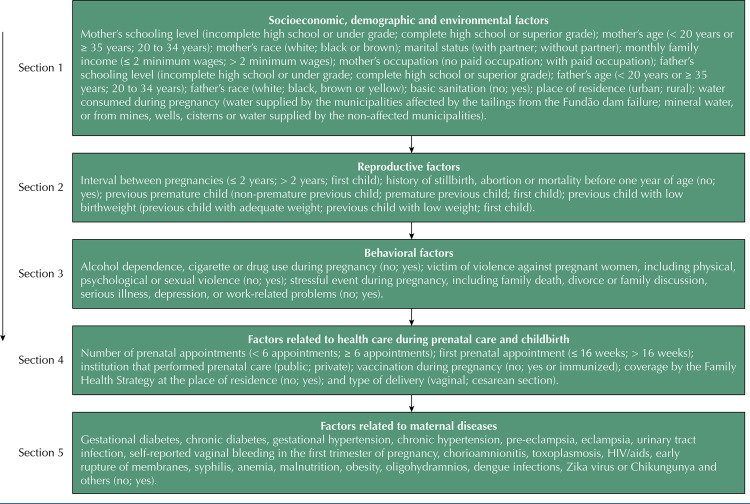
Explanatory model of the independent variables divided into blocks and order of input of the factors in the logistic regression analysis.

Logistic regression analysis was used to verify the associations of independent variables with low birthweight. The associated factors that presented p-value below 0.20 in the bivariate analysis per block were considered eligible to compose the multivariate models. Logistic regression analysis was performed according to the order of entry: first the socioeconomic, demographic and environmental factors (block 1), followed by reproductive factors (block 2), behavioral factors (block 3), factors related to health care during prenatal care and childbirth (block 4) and, finally, factors related to maternal diseases (block 5), according to Figure 1. The previously selected variables were submitted to the new multivariate analysis, using p-value below 0.05 as a parameter for permanence in the final model. The research was approved by the Ethics and Research Committee on Human Beings (CAAE 61055716.4.0000.5147), being conducted within the required ethical standards.

## RESULTS

The study included 65 live births belonging to the case group and 130 to the control group. We excluded a case of low birthweight with a diagnosis of genetic syndrome and 18 cases that did not have their respective controls. Nine puerperal women did not agree to participate.

Of the participant live births, 118 (60.5%) had mothers living in Governador Valadares and 23 (11.8%) in other eight neighboring municipalities that were also hit by the mud. The remaining 54 live births (27.7%) had mothers who lived in neighboring municipalities not affected by the disaster.

There was a predominance of mineral water consumption during pregnancy (38.5%), but 23% of the puerperal women consumed water from a well, mine or cistern while pregnant. The others consumed the water supplied by the municipality of Governador Valadares (14.9%), from the other municipalities affected by mud (7.7%) and unaffected municipalities (15.9%). The only variable, among the socioeconomic, demographic and environmental factors, that presented a significant association with low birthweight in the bivariate analysis was “water consumed during pregnancy” (p = 0.008), as observed in Table 1 .

**Table 1:** Number of cases and controls, odds ratio, 95% confidence interval and p-value for socioeconomic, demographic and environmental factors (section 1).

Variables	Cases (n = 65)		Controls (n = 130)		OR	95%CI	p
	
	n	%	n	%			
Mother’s schooling level							
Incomplete high school or under grade	32	49.2	63	48.5	Ref		
Complete high school or superior grade	33	50.8	67	51.5	0.970	0.535–1.759	0.919
Mother’s age							
< 20 years or ≥ 35 years	17	26.2	40	30.8	Ref	-	
20 to 34 years	48	73.8	90	69.2	1.255	0.644–2.445	0.504
Mother’s race							
White	11	16.9	18	13.8	Ref	-	
Black or brown	54	83.1	112	86.2	0.789	0.348–1.787	0.569
Mother’s marital status							
With a partner	49	75.4	101	77.7	Ref	-	
Without partner	16	24.6	29	22.3	1.137	0.565–2.288	0.718
Monthly household income^b^							
≤ 2 minimum wages	48	78.7	93	75.0	Ref	-	
> 2 minimum wages	13	21.3	31	25.0	0.813	0.389–1.695	0.580
Mother’s occupation							
No paid occupation	42	64.6	84	64.6	Ref	-	
With paid occupation	23	35.4	46	35.4	1.000	0.537–1.864	1.000
Father’s schooling level							
Incomplete high school or under grade	32	55.2	69	59.0	Ref	-	
Complete high school or superior grade	26	44.8	48	41.0	1.168	0.619–2.204	0.632
Father’s age^b^							
< 20 years or ≥ 35 years	15	24.2	37	28.7	Ref	-	
20 to 34 years	47	75.8	92	71.3	1.260	0.629–2.526	0.514
Father’s race							
White	18	27.7	32	24.6	Ref	-	
Black, brown or yellow	47	72.3	98	75.4	0.853	0.435–1.673	0.643
Basic sanitation							
No	10	15.4	12	9.2	Ref	-	
Yes	55	84.6	118	90.8	0.559	0.228–1.373	0.200
Place of residence							
Urban	50	76.9	105	80.8	Ref	-	
Rural	15	23.1	25	19.2	1.260	0.611–2.597	0.531
Water consumed during pregnancy							
Mineral, from mines, wells, cisterns or the supply service of non-affected municipalities	43	66.2	108	83.1	Ref	-	0.008^a^
Supply services of affected municipalities	22	33.8	22	16.9	2.512	1.262–5.00	

RC: odds ratio; 95%CI: 95% confidence interval; p-value: level of statistical significance; Ref: reference category

^a^ p < 0.20

^b^ Some puerperal women did not know or did not agree to report the family’s monthly income (n = 10), father’s schooling level (n = 20) and father’s age (n = 4), and these data were considered missing.

Regarding reproductive factors, the variable “previous birth of underweight child” (p = 0.065) presented p < 0.20, as well as the variables “cigarette use” (p = 0.036) and “victim of violence against pregnant women” (p = 0.140), referring to behavioral factors ( Table 2 ).

**Table 2:** Number of cases and controls, odds ratio, 95% confidence interval and p-value of reproductive (block 2) and behavioral (block 3) factors.

Variables	Cases (n = 65)		Controls (n = 130)		OR	95%CI	p
	
	n	%	n	%			
Section 2							

Interval between pregnancies							
≤ 2 years	5	7.7	15	11.5	Ref	-	0.302
2 years	21	32.3	52	40.0	1.212	0.391–3.758	0.740
First child	39	60.0	63	48.5	1.857	0.626–5.513	0.265
Previous abortion							
No	54	83.1	110	84.6	Ref	-	
Yes	11	16.9	20	15.4	1.120	0.501–2.505	0.782
Previous case of stillbirth							
No	64	98.5	128	98.5	Ref	-	
Yes	1	1.5	2	1.5	1.000	0.089–11.236	1.000
Child’s death in under 1 year							
No	64	98.5	126	96.9	Ref	-	
Yes	1	1.5	4	3.1	0.492	0.54–4.495	0.666
Previous premature child							
Non-premature previous child	25	38.5	60	46.2	Ref	-	0.218
Premature previous child	1	1.5	7	5.4	0.343	0.040–2.933	0.328
First child	39	60.0	63	48.5	1.486	0.804–2.746	0.206
Previous child with low birthweight							
Previous child with adequate weight	21	32.3	63	48.5	Ref	-	0.063*
Previous child with low weight	5	7.7	4	3.1	3.750	0.921–15.275	0.065*
First child	39	60.0	63	48.5	1.857	0.984–3.505	0.056*

Section 3							

Alcohol dependence							
No	58	89.2	119	91.5	Ref	-	
Yes	7	10.8	11	8.5	1.306	0.481–3.543	0.600
Smoking habit							
No	55	84.6	122	93.8	Ref	-	
Yes	10	15.4	8	6.2	2.773	1.038–7.408	0.036*
Drug use							
No	64	98.5	129	99.2	Ref	-	
Yes	1	1.5	1	0.8	2.016	0.124–32.750	1.000
Victim of violence against pregnant women							
No	57	87.7	122	93.8	Ref	-	
Yes	8	12.3	8	6.2	2.140	0.765–5.990	0.140*
Stressful event in pregnancy							
No	40	61.5	89	68.5	Ref	-	
Yes	25	38.5	41	31.5	1.357	0.729–2.526	0.335

RC: odds ratio; 95%CI: 95% confidence interval; p: level of statistical significance; Ref: reference category

* p < 0.20

Only one puerperal woman, belonging to the case group, did not perform prenatal care and thus did not attend any appointment; three attended only one appointment and, in total, 45 puerperal women attended less than six appointment. As the sample would be insufficient for the analysis using groups with a smaller number of appointments, we chose to classify this variable into only two categories, as described above. In this section, referring to factors related to health care during prenatal care and childbirth, no variable presented a significant association with low birthweight ( Table 3 ).

**Table 3:** Number of cases and controls, odds ratio, 95% confidence interval and p-value of factors related to health care during prenatal care and childbirth (section 4).

Variables	Cases (n = 65)		Controls (n = 130)		OR	95%CI	p
	
	n	%	n	%			
Number of prenatal appointments							
< 6 appointments	18	27.7	27	20.8	Ref	-	
≥ 6 appointments	47	72.3	103	79.2	0.684	0.344–1.363	0.279
First prenatal appointment*							
≤ 16 weeks	52	81.3	105	80.8	Ref	-	
> 16 weeks	12	18.8	25	19.2	0.969	0.451–2.082	0.936
Pre-natal care institution*							
Public	54	84.4	103	79.2	Ref	-	
Private	10	15.6	27	20.8	0.706	0.318–1.567	0.391
Vaccination during pregnancy							
No	4	6.2	10	7.7	Ref	-	
Yes, or immunized	61	93.8	120	92.3	1.271	0.383–4.218	0.695
Residence with FHS coverage							
No	5	7.7	7	5.4	Ref	-	
Yes	60	92.3	123	94.6	0.683	0.208–2.241	0.527
Type of delivery							
Vaginal	40	61.5	89	68.5	Ref	-	
Cesarean section	25	38.5	41	31.5	1.357	0.729–2.526	0.335

FHS: Family Health Strategy RC: odds ratio; 95%CI: 95% confidence interval; p: level of statistical significance; Ref: reference category

* A participating puerperal woman did not perform prenatal care and was considered absent in the analysis of the data of the first prenatal and prenatal appointment.

Finally, 145 puerperal women presented some form of maternal disease during pregnancy. In this section, the variables that presented p < 0.20 were selected: “urinary tract infection” (p = 0.089) and “self-reported vaginal bleeding in the first trimester of pregnancy” (p = 0.112), as shown in Table 4 .

**Table 4:** Number of cases and controls, odds ratio, 95% confidence interval and p-value of factors related to maternal diseases (section 5).

Variables	Cases (n = 65)		Controls (n = 130)		OR	95%CI	p
	
	n	%	n	%			
Gestational diabetes							
No	63	96.9	126	96.9	Ref	-	
Yes	2	3.1	4	3.1	1.000	0.178–5.608	1.000
Gestational hypertension							
No	54	83.1	114	87.7	Ref	-	
Yes	11	16.9	16	12.3	1.451	0.631–3.339	0.379
Chronic hypertension							
No	63	96.9	129	99.2	Ref	-	
Yes	2	3.1	1	0.8	4.095	0.364–46.022	0.258
Preeclampsia							
No	62	95.4	128	98.5	Ref	-	
Yes	3	4.6	2	1.5	3.097	0.504–19.012	0.336
Urinary tract infection							
No	37	56.9	90	69.2	Ref	-	
Yes	28	43.1	40	30.8	1.703	0.919–3.153	0.089*
Vaginal bleeding in the 1st trimester							
No	52	80.0	115	88.5	Ref	-	
Yes	13	20.0	15	11.5	1.917	0.851–4.316	0.112*
Oligohydramnios							
No	62	95.4	125	96.2	Ref	-	
Yes	3	4.6	5	3.8	1.210	0.280–5.226	1.000
Anemia							
No	49	75.4	91	70.0	Ref	-	
Yes	16	24.6	39	30.0	0.762	0.387–1.500	0.431
Syphilis							
No	63	96.9	125	96.2	Ref	-	
Yes	2	3.1	5	3.8	0.794	0.150–4.206	1.000

RC: odds ratio; 95%CI: 95% confidence interval; p: level of statistical significance; Ref: reference category

* p < 0.20

In the logistic regression analysis, the first introduced were variables “water consumed during pregnancy” (section 1) and “previous case of low birthweight” (section 2), both of which were maintained in the model. Then we introduced variables of section 3, “cigarette use” and “victim of violence against pregnant women,” with later removal of the latter due to loss of significance. As section 4 did not present any variable with p < 0.20, the variables of section 5 were added: “urinary tract infection” and “vaginal bleeding.” These last two variables were also removed from the analysis because they did not present a significant association with low birthweight.

Table 5 shows the result of the final logistic regression model, which indicates they presented a significant association with low birthweight: first child (odds ratio [OR] = 2.033; p = 0.036), smoking during pregnancy (OR = 2.850; p = 0.047) and water provided by the municipalities affected by the mud (OR = 2.444; p = 0.013).

**Table 5:** Result of logistic regression (odds ratio, 95% confidence interval and p-value) of factors associated with low birthweight.

Sections	Variables	OR	95%CI	p
Section 1	Water consumed during pregnancy			
	Mineral, from mines, wells, cisterns or the supply service of non-affected municipalities	Ref	-	
	Supply services of affected municipalities	2.444	1.203–4.965	0.013*

Section 2	Previous child with low birthweight			
	Previous child with adequate weight	Ref	-	0.052
	Previous child with low weight	3.729	0.860–16.167	0.079
	First child	2.033	1.047–3.948	0.036*

Section 3	Smoking habit			
	No	Ref	-	
	Yes	2.850	1.013–8.021	0.047*

RC: odds ratio; 95%CI: 95% confidence interval; p-value: level of statistical significance; Ref: reference category

* p < 0.05.

## DISCUSSION

After the analysis adjusted for the other factors under study, we found that the higher risks of low birthweight are associated with the first children and live births whose mothers smoked during pregnancy and consumed the water supplied by the municipalities affected by the tailings from the Fundão dam failure. One of the possible explanations for the result regarding the water consumed during pregnancy may be related to the water pollution of Rio Doce caused by one of the greatest socio-environmental tragedies in the country. On November 5, 2015, in Mariana, Minas Gerais, more than 70 million cubic meters of iron ore tailings sludge leaked after a Samarco dam collapsed, a joint venture of Brazil’s Vale and Anglo-Australian BHP Billiton^11^ . In addition to destroying villages, the mud traveled 663 km along the rivers Doce, Gualaxo do Norte and Carmo, reaching 35 municipalities in Minas Gerais and four in Espírito Santo^11 , 12^ .

According to Wanderley et al.^12^ , recent studies have presented varied evidence on the presence of heavy metals in the river and previous studies have already shown the contamination of the river by metals, due to mineral processing in the upper Rio Doce. The presence of these metals puts the population’s long-term health at risk, with the possibility of a considerable increase in chronic diseases^12^ . Many questions are still being raised about what levels of contamination and health effects are present and expected in the exposed population, especially in soil and water quality for human consumption^11^ . The population of Governador Valadares seems to fear the contamination of the water of the Rio Doce, which was once again supplied for human consumption. This situation may explain the fact that almost 40% of the families participating in this study still use mineral water for consumption, two to three years after the disaster. Financial expenses with the purchase of mineral water aggravate the situation, since the population studied presented predominantly low economic level, with monthly family income less than two minimum wages.

Newborns whose mothers, during pregnancy, consumed water provided by municipalities affected by the mud had a higher risk of low birthweight compared to those whose mothers consumed mineral water, from wells, mines, cisterns or supplied by unaffected municipalities. Bezerra^13^ evaluated the concentration of some metals in the water available for consumption in Governador Valadares, through the analysis of samples from five collection points, and compared it with the specific legislation on potability. According to the results, there were elevated levels of aluminum, selenium and antimony in the samples, making the water unfit for consumption according to the potability criterion.

Public authorities of the state of Minas Gerais evaluated the quality of water distributed by the supply service of Governador Valadares to residents of several neighborhoods of the city. The results of the analyses, carried out in 2016, showed that no toxic and harmful metals were detected at concentrations higher than potability standards. However, aluminum presented concentrations above the limits established by the Consolidation Ordinance of the Ministry of Health No. 5, of September 28, 2017. In addition, at some collection points, the total coliform and turbidity parameters of the water did not meet the potability criteria^14^ . Considering that dam tailings have high concentrations of aluminum in their composition, it is possible that this metal was transported along the Rio Doce, causing changes in the chemical composition in various stretches of the watercourse, according to the direction of the winds, rainfall indices and river flow. In addition, it was found in the evaluation that only aluminum sulfate has been used as a coagulant for water treatment, and the use of the black acacia polymer was interrupted^14 , 15^ . Aluminum salts are widely used as coagulants in the drinking water treatment process; however, its use may increase the concentration of this metal in treated waters^16^ . Thus, the presence of residual aluminum concentrations detected in the water intended for the supply of the population may have as its origin not only the existence of this element in the uptake, but also in the use of coagulant based on aluminum salts, in the treatment process and in the operating conditions of the water treatment plant^14 , 15^ .

The mother’s environment has implications for the health of the fetus, which is transplacentally exposed to contaminants present in food, water and air^17^ . Some contaminants can harm both the mother and fetus, such as aluminum. Humans are naturally exposed to aluminum through drinking water, food, medicines, dust, hygiene products or cooking utensils^17^ . Despite the knowledge about aluminum toxicity, little is known about its effects in humans, and most studies are conducted in models with laboratory animals. It is known, however, that fetuses, newborns and developing infants, due to their immaturity, are more vulnerable to the toxicity of exposure to this metal^18 , 19^ .

A study conducted to evaluate the effect of exposure to aluminum, administered in drinking water, in rats during pregnancy, lactation and post-weaning, observed that rats exposed to this component gained less weight during pregnancy and consumed less water and food during the lactation period, and their offspring had decreased body weight in comparison to the control group^20^ . Aluminum ingested during pregnancy also affects the metabolism of essential elements such as calcium, magnesium, manganese, copper, zinc and iron^19^ . A study conducted with pregnant rats treated with aluminum showed that oral exposure to this metal during pregnancy can produce significant changes in the tissue distribution of several essential elements, with possible consequences on the metabolism of the fetus^21^ .

Another explanation for the results is the possibility of pesticides in the water consumed by the population. Data from the Ministry of Health and the Human Consumption Water Quality Surveillance Information System show that different pesticides were found in the water of 25% of municipalities in Brazil between 2014 and 2017. Twenty-seven types of pesticides were tested, 16 of which are classified as highly toxic and 11 are associated with diseases such as fetal malformation and cancer. In Governador Valadares, 27 pesticides were detected in the water that supplies the population, but none with concentration higher than that allowed by Brazilian legislation. However, 24 of these pesticides are concentrated above the safe limit set by the European Union^22^ . One of them was atrazine, which is present as a mixture in drinking water, groundwater and surface water and can alter fetal growth. A cohort study conducted in France evaluated the association between atrazine exposure during pregnancy and adverse effects at birth, using urine samples, and found a relationship with fetal growth restriction (OR = 1.5; 95% confidence interval [95%CI] = 1.0–2.2). The authors point out that atrazine was banned in Europe and that the study shows the persistence of this component in the environment, alerting countries in which it is still in use, such as Brazil, the United States, Argentina, Mexico and China^23^ .

Newborns who were the first children of their mothers had a higher risk of low birthweight compared with those whose mothers had previous children with adequate weight. The biological mechanisms of how parity can influence the incidence of low birthweight is not yet well known. A systematic review and meta-analysis that investigated the risks in pregnancy for the different parities found a significant reduction (280 grams) in birthweight in first children in relation to women who have already had two to four other births^24^ . A study conducted in Indonesia detected a 46% higher risk (OR = 1.46; p = 0.030) of having children with low birthweight in women pregnant for the first time compared with those who had one previous pregnancy^25^ . The low birthweight rate tends to be higher in the first child than in the second and third^26^ . This can be explained by the fact that maturation of uterine structures occur in the first pregnancy, which improves their conditions and allows for greater placental development and better fetal nutrition^27^ .

Smoking during pregnancy is also noteworthy as it presents risks to the newborn. A systematic review and meta-analysis investigated the relationship between maternal smoking and low birthweight and found that pregnant smokers were twice as likely to have a child with the condition when compared with nonsmokers^28^ . Zhang et al.^29^ found that infants whose mothers smoked during pregnancy had the three anthropometric measurements at birth (weight, head circumference, and length) reduced. In view of the high prevalence and adverse effects on fetal nervous system development and infant morbidity and mortality, it is necessary to intervene in order to reduce smoking among pregnant women. As the gestational period is the most appropriate to encourage smoking cessation, its harmful effects on the health of pregnant women and the fetus should be reinforced in prenatal appointments by the health team more intensely and repeatedly^30^ .

One of the limitations of this study is the sample size. However, all mothers of live births who met the inclusion criteria were invited to participate. The greatest difficulty was due to the pairing criteria adopted. Pairing by sex was necessary due to greater vulnerability and association with perinatal and neonatal mortality in boys^31^ . In addition, the literature reports a higher risk of low birthweight in girls^25^ , in agreement with what was found in this study, as 50.8% of newborns were girls. Pairing through date of birth was conducted to make the sample as similar as possible regarding perinatal assistance, including human resources and professional qualification of the working staff.

Another limitation is that this study did not assess water quality, using only information about the type of water consumed during pregnancy. To this moment, few were the publications that assess the quality of the water consumed by the population of Governador Valadares and neighboring municipalities^13 , 15^ , which hinders general results. Furthermore, there are no studies in the region previous to the disaster, which makes it impossible to state that it was responsible for the contamination profile observed^12 , 32^_._ Finally, we did not assess the exposure of pregnant women to the aluminum in food, air, kitchen utensils and hygiene items and thus we could not make sure that the metal was present only in the consumed water.

We can conclude among the analyzed factors that water consumed during pregnancy, first pregnancy and smoking habits presented association with low birthweight in the study population. We reinforce the importance of epidemiological studies that assess water quality and its hazards, as well as the need to greater control in the prenatal period of women pregnant for the first time and greater support of anti-smoking policies, specially during pregnancy. Further studies in the field are needed, as well as the periodic assessment of water quality.

## References

[1] 1. González-Jiménez J, Rocha-Buelvas A. Risk factors associated with low birth weight in the Americas : literature review. Rev Fac Med Univ Nac Colomb. 2018;66(2):255-60. 10.15446/revfacmed.v66n2.61577

[2] 2. United Nations Children’s Fund; World Health Organization. Low birthweight: country, regional and global estimates. New York: UNICEF; 2004.

[3] 3. Fondo de las Naciones Unidas para la Infancia. Examen estadístico de un mundo apropiado para los niños y las niñas. Nueva York: UNICEF; 2007. (Progreso para la infancia, 6).

[4] 4. Buriol VCS, Hirakata V, Goldani MZ, Silva CH. Temporal evolution of the risk factors associated with low birth weight rates in Brazilian capitals (1996-2011). Popul Health Metr. 2016;14:15 10.1186/s12963-016-0086-0 PMC4855447 27147908

[5] 5. Leal MC, Szwarcwald CL, Almeida PVB, Aquino EML, Barreto MC, Barros F, et al. Reproductive, maternal, neonatal and child health in the 30 years since the creation of the Unified Health System (SUS). Cienc Saude Coletiva. 2018;23(6):1915-28. 10.1590/1413-81232018236.03942018 29972499

[6] 6. Slovic AD, Diniz CS, Ribeiro H. Clean air matters: an overview of traffic-related air pollution and pregnancy. Rev Saude Publica. 2017;51:5. 10.1590/s1518-8787.2017051006652 PMC5308554 28225911

[7] 7. Gouveia N, Bremner SA, Novaes HMD. Association between ambient air pollution and birth weight in São Paulo, Brazil. J Epidemiol Community Health. 2004;58(1):11-7. 10.1136/jech.58.1.11 PMC1757020 14684720

[8] 8. Ministério da Saúde (BR). Programa Humanização do Parto: humanização do pré-natal e nascimento. Brasília (DF); 2002 [cited 2019 Out 19]. Available from: http://bvsms.saude.gov.br/bvs/publicacoes/parto.pdf

[9] 9. Viellas EF, Domingues RMSM, Dias MAB, Gama SGN, Theme-Filha MM, Costa JV, et al. Assistência pré-natal no Brasil. Cad Saude Publica. 2014;30 Supl 1:S85-100. 10.1590/0102-311X00126013

[10] 10. Mayfield D, McLeod G, Hall P. The CAGE Questionnaire: validation of a new alcoholism screening instrument. Am J Psychiatry. 1974;131(10):1121-3. 10.1176/ajp.131.10.1121 4416585

[11] 11. Porto MFS. A tragédia da mineração e do desenvolvimento no Brasil: desafios para a saúde coletiva. Cad Saude Publica. 2016;32(2):e00211015. 10.1590/0102-311X00211015

[12] 12. Wanderley LJ, Mansur MS, Milanez B, Pinto RG. Desastre da Samarco/Vale/BHP no Vale do Rio Doce: aspectos econômicos, políticos e socio ambientais. Cienc Cult. 2016;68(3):30–5. 10.21800/2317-66602016000300011

[13] 13. Bezerra ES. Determinação de metais na água disponibilizada para consumo humano no município de Governador Valadares - MG. Brasília, DF: Universidade de Brasília, Faculdade de Ceilândia; 2016. Trabalho de Conclusão de Curso (graduação) de Farmácia.

[14] 14. Diniz PS. Inquérito civil n°0105.15.002048-2. Qualidade da água no município de Governador Valadares após o desastre ambiental causado pelo rompimento da barragem da Samarco Mineração S/A. Belo Horizonte: Ministério Público do Estado de Minas Gerais; Procuradoria Geral de Justiça; 2016. (Laudo IDCEAT 26935311).

[15] 15. Diniz PS. Requerimento de apoio técnico formulado pelas 10ª e 14ª Promotorias de Justiça da Comarca de Governador Valadares. Belo Horizonte: Ministério Público do Estado de Minas Gerais; Procuradoria Geral de Justiça; 2018. (Laudo SISCEAT32636944).

[16] 16. Wang W, Yang H, Wang X, Jiang J, Zhu W. Effects of fulvic acid and humic acid on aluminum speciation in drinking water. J Environ Sci. 2010;22(2):211-7. 10.1016/S1001-0742(09)60095-4 20397408

[17] 17. Katic J, Fucic A, Gamulin M. Prenatal, early life, and childhood exposure to genotoxicants in the living environment. Arch Ind Hyg Toxicol. 2010;61(4):455-64. 10.2478/10004-1254-61-2010-2065 21183437

[18] 18. Röllin HB, Nogueira C, Olutola B, Channa K, Odland J. Prenatal exposure to aluminum and status of selected essential trace elements in rural South African women at delivery. Int J Environ Res Public Health. 2018;15(7):1494. 10.3390/ijerph15071494 PMC6068832 30011954

[19] 19. Fanni D, Ambu R, Gerosa C, Nemolato S, Iacovidou N, Van Eyken P, et al. Aluminum exposure and toxicity in neonates: a practical guide to halt aluminum overload in the prenatal and perinatal periods. World J Pediatr. 2014;10(2):101-7. 10.1007/s12519-014-0477-x 24801228

[20] 20. Colomina MT, Roig JL, Torrente M, Vicens P, Domingo JL. Concurrent exposure to aluminum and stress during pregnancy in rats: effects on postnatal development and behavior of the offspring. Neurotoxicol Teratol. 2005;27(4):565-74. 10.1016/j.ntt.2005.06.014 16024221

[21] 21. Bellés M, Albina ML, Sanchez DJ, Corbella J, Domingo JL. Effects of oral aluminum on essential trace elements metabolism during pregnancy. Biol Trace Elem Res. 2001;79(1):67-81. 10.1385/BTER:79:1:67 11318238

[22] 22. Aranha A, Rocha L. 1 em 4 municípios tem “coquetel” com agrotóxicos na água. Exame. 2019 Apr 17 [updated 2019 Jun 13; cited 2019 Jun 15]. Available from: https://exame.abril.com.br/brasil/1-em-4-municipios-tem-coquetel-com-agrotoxicos-na-agua-consulte-o-seu/

[23] 23. Chevrier C, Limon G, Monfort C, Rouget F, Garlantézec R, Petit C, et al. Urinary biomarkers of prenatal atrazine exposure and adverse birth outcomes in the PELAGIE Birth Cohort. Environ Health Perspect. 2011;119(7):1034-41. 10.1289/ehp.1002775 PMC3222984 21367690

[24] 24. Shah SS; Knowledge Synthesis Group on Determinants of LBW/PT Births. Parity and low birth weight and preterm birth: a systematic review and meta-analyses. Acta Obstet Gynecol Scand. 2010;89(7):862-75. 10.3109/00016349.2010.486827 20583931

[25] 25. Andayasari L, Opitasari C. Parity and risk of low birth weight infant in full term pregnancy. Health Sci J Indones. 2016;7(1):13-6. 10.22435/hsji.v7i1.4701.13-16

[26] 26. Oh Y, Bae J. Impact of changes in maternal age and parity distribution on the increasing trends in the low birth weight and very low birth weight rates in South Korea, 2005-2015. J Prev Med Public Health. 2019;52(2):123-30. 10.3961/jpmph.18.247 PMC6459761 30971079

[27] 27. Valero de Bernabé J, Soriano T, Albaladejo R, Juarranz M, Calle ME, Martínez D, et al. Risk factors for low birth weight: a review. Eur J Obstet Gynecol Reprod Biol. 2004;116(1):3-15. 10.1016/j.ejogrb.2004.03.007 15294360

[28] 28. Pereira PPS, Mata FAF, Figueiredo ACG, Andrade KRC, Pereira MG. Maternal active smoking during pregnancy and low birth weight in the Americas: a systematic review and meta-analysis. Nicotine Tob Res. 2017;19(5):497-505. 10.1093/ntr/ntw228 28403455

[29] 29. Zhang L, González-Chica DA, Cesar JA, Mendoza-Sassi RA, Beskow B, Larentis N, et al. Tabagismo materno durante a gestação e medidas antropométricas do recém-nascido: um estudo de base populacional no extremo sul do Brasil. Cad Saude Publica. 2011;27(9):1768-76. 10.1590/S0102-311X2011000900010 21986604

[30] 30. Dias-Damé JL, Lindsay AC, Cesar JA. Cessação do tabagismo na gestação: estudo de base populacional. Rev Saude Publica. 2019;53:3. 10.11606/S1518-8787.2019053000619

[31] 31. Chiavegatto Filho ADP, Laurenti R. O sexo masculino vulnerável: razão de masculinidade entre os óbitos fetais brasileiros. Cad Saude Publica. 2012;28(4):720-8. 10.1590/S0102-311X2012000400011. 22488317

[32] 32. Carvalho GO, Pinheiro ADA, Sousa DM, Padilha JA, Souza JS, Galvão PM, et al. Metals and arsenic in water supply for riverine communities affected by the largest environmental disaster in Brazil: The Dam Collapse on Doce River. Orbital Electron J Chem. 2018;10(4 Spec Nº). 10.17807/orbitalv10i4.1081

