# Supplementary material for: Factors associated with low birthweight: a case-control study in a city of Minas Gerais
Source: Rev Saude Publica. 2020 Jul 15;54:71. doi: 10.11606/s1518-8787.2020054002065 (PMC7362952; doi:10.11606/s1518-8787.2020054002065)
Supplement: Translation

# Translation: Fatores associados ao baixo peso ao nascer: estudo caso-controle em cidade de Minas Gerais

**Authors:** Érica Cesário Defilipo, Paula Silva de Carvalho Chagas, Andreia Peraro-Nascimento, Luiz Cláudio Ribeiro

**Affiliations:** I Brasil Universidade Federal de Juiz de Fora campus Governador Valadares. Instituto de Ciências da Vida. Departamento de Fisioterapia. Governador Valadares, MG, Brasil; II Brasil Universidade Federal de Juiz de Fora. Faculdade de Fisioterapia. Programa de Pós-graduação em Ciências da Reabilitação e Desempenho Físico-funcional. Juiz de Fora, MG, Brasil; III Brasil Universidade Federal de Juiz de Fora campus Governador Valadares. Instituto de Ciências da Vida. Departamento de Farmácia. Governador Valadares, MG, Brasil; IV Brasil Universidade Federal de Juiz de Fora. Departamento de Estatística. Programa de Pós-Graduação em Saúde Coletiva. Juiz de Fora, MG, Brasil

**Keywords:** Recém-Nascido de Baixo Peso, Fatores de Risco, Exposição Ambiental, Fatores Socioeconômicos, Estudos de Casos e Controles, Saúde Materno-Infantil

## Abstract

**OBJETIVO:**

Analisar os fatores socioeconômicos, demográficos, ambientais, reprodutivos, comportamentais, de assistência à saúde, doenças maternas e, sobretudo, o possível impacto do desastre ambiental ocorrido em Mariana, pela exposição pré-natal a diferentes fontes de água para consumo humano, associados ao baixo peso ao nascer em nascidos vivos a termo no Hospital Municipal de Governador Valadares, Minas Gerais.

**MÉTODOS:**

Estudo caso-controle, realizado com nascidos vivos no Hospital Municipal de Governador Valadares, no período de maio de 2017 a julho de 2018. O grupo caso foi composto por nascidos vivos a termo e baixo peso ao nascer e o grupo controle, por nascidos vivos a termo e com peso adequado, pareados por sexo e data de nascimento. Para cada caso foram selecionados dois controles. A coleta de dados foi realizada por meio de entrevista com as puérperas e informações complementares foram obtidas pela análise do cartão de pré-natal e prontuários. Para análise dos dados, foi realizada regressão logística.

**RESULTADOS:**

Participaram do estudo 65 nascidos vivos pertencentes ao grupo caso e 130 ao grupo controle. Após a análise ajustada para os demais fatores em estudo, verificou-se que os riscos mais elevados de baixo peso ao nascer estão associados aos primeiros filhos (RC = 2,033; IC95% = 1,047–3,948; p = 0,036) e aos nascidos vivos cujas mães utilizaram cigarro durante a gestação (RC = 2,850; IC95% = 1,013–8,021; p = 0,047) e consumiram a água fornecida pelos serviços de abastecimento dos municípios atingidos pelos rejeitos provenientes do rompimento da barragem de Fundão (RC = 2,444; IC95% = 1,203–4,965; p = 0,013).

**CONCLUSÕES:**

A água consumida na gestação, primiparidade e tabagismo materno apresentaram associação com baixo peso ao nascer na população estudada. Reforça-se a importância de estudos epidemiológicos, que avaliem a qualidade da água e seus efeitos adversos na saúde, assim como maior controle no pré-natal das gestantes que terão o primeiro filho e maior apoio das políticas contra o tabagismo, especialmente durante a gravidez.

## INTRODUÇÃO

A cada ano nascem, em todo o mundo, mais de vinte milhões de crianças com baixo peso (BP) ao nascer, que é definido como peso inferior a 2.500 gramas^1 , 2^ . O BP ao nascer é considerado como um importante marcador de risco para mortalidade infantil, além de contribuir para morbidades como doenças infecciosas, alterações cognitivas e neurológicas, atraso do desenvolvimento e maior probabilidade de desenvolver doenças crônicas^3^ .

As taxas de BP ao nascer no Brasil, considerando a análise do período de 1996 a 2011, mostram estabilidade de cerca de 8%. Analisando as regiões do país, as maiores taxas foram encontradas nas regiões mais desenvolvidas, Sul e Sudeste, e aumento significativo foi observado nas regiões menos desenvolvidas (Norte, Nordeste e Centro-Oeste)^4^ . As razões para o aumento dessas taxas, considerando a melhora dos indicadores sociais e de saúde materno-infantil nos últimos anos, ainda não são bem conhecidas^5^ .

O BP ao nascer é considerado um complexo problema de saúde pública por sua etiologia multifatorial. Um estudo de revisão sobre o BP ao nascer nas Américas mostrou que a maioria dos trabalhos publicados nos últimos anos, concordam com a associação de fatores sociodemográficos, biológicos e comportamentais. Estudos que referem a associação do BP ao nascer com fatores de risco ambientais, como a exposição à poluição atmosférica, da água ou do solo, estão ganhando cada vez mais importância^1^ .

A associação do BP ao nascer com a poluição do ar já foi relatada na literatura^1 , 6^ , mas poucos estudos avaliaram a relação desse desfecho com a qualidade da água. Diante das tragédias socioambientais ocorridas em Minas Gerais, como o rompimento da barragem de Fundão em Mariana, tornam-se necessárias pesquisas que avaliem a qualidade da água e seus efeitos adversos na saúde. Em algumas fases da vida, os danos causados à saúde pela poluição podem ser irreversíveis. Tal situação torna-se mais grave quando nos referimos à saúde materno-infantil, devido à interferência na reprodução e nas condições gestacionais e de vida do concepto^7^ .

Este estudo objetivou analisar os fatores socioeconômicos, demográficos, ambientais, reprodutivos, comportamentais, de assistência à saúde, doenças maternas e, sobretudo, o possível impacto do desastre ambiental ocorrido em Mariana, pela exposição pré-natal a diferentes fontes de água para consumo humano, associados ao baixo peso ao nascer em nascidos vivos a termo no Hospital Municipal de Governador Valadares, Minas Gerais.

## MÉTODOS

Estudo caso-controle, realizado com nascidos vivos no Hospital Municipal de Governador Valadares, no período de maio de 2017 a julho de 2018, cujas mães residiam nesse município ou na região. Esse hospital foi escolhido por atender ao Sistema Único de Saúde e por ser o único que possui unidade de terapia intensiva neonatal na região, sendo considerado referência para as cidades do vale do rio Doce. Foram considerados casos os nascidos vivos a termo, com idade gestacional igual ou superior a 37 semanas e inferior a 42 semanas e com peso inferior a 2.500 gramas (baixo peso ao nascer), enquanto os controles foram os nascidos vivos a termo com peso igual ou superior a 2.500 gramas (peso adequado ao nascer), pareados pelo sexo e data de nascimento. Para cada caso foram selecionados dois controles. Foram excluídos do estudo nascidos vivos com malformações congênitas, síndromes genéticas, doenças progressivas e lesões do sistema nervoso, diagnosticadas ou suspeitas ao nascimento. Quando existiam mais de dois recém-nascidos que atendiam aos critérios de inclusão para compor o grupo controle, foi realizado sorteio. Quando não existiam dois controles para o pareamento, o caso não era incluído no estudo.

A coleta de dados foi realizada por meio de entrevista estruturada com as puérperas, ainda no período de internação hospitalar, em um prazo de 24 a 48 horas após o parto. Informações complementares foram obtidas pela análise do cartão de pré-natal e dos prontuários da puérpera e do recém-nascido. Os dados foram coletados por pesquisadores previamente treinados.

Foi considerado como variável dependente o BP ao nascer. As variáveis independentes foram organizadas em cinco blocos, de acordo com o modelo explicativo apresentado na Figura 1 , na qual consta a forma de categorização de cada variável estudada. Para analisar a variável “água consumida durante a gestação”, levou-se em consideração que os participantes residiam em municípios diferentes, alguns atingidos pelos rejeitos provenientes do rompimento da barragem de Fundão e outros não. Por esse motivo essa variável foi categorizada em: 1) água mineral, mina, poço ou cisterna ou água proveniente do serviço de abastecimento de água dos municípios não atingidos pela lama; e 2) água proveniente do serviço de abastecimento de água dos municípios atingidos. A categorização das variáveis “número de consultas de pré-natal” e “primeira consulta de pré-natal” foi definida com base no proposto pelo Ministério da Saúde, que determina o início da assistência pré-natal até a décima sexta semana de gestação e o mínimo de seis consultas^8^ . A variável “número de consultas” foi categorizada de acordo com o que seria preconizado (seis consultas ou mais) ou não preconizado (menos de seis consultas) e a variável “primeira consulta de pré-natal” foi categorizada como até 16 semanas e mais de 16 semanas^9^ ( Figura 1 ). A detecção da dependência do álcool foi realizada por meio do instrumento CAGE (acrônimo para *cut down, annoyed, guilty* e *eye-opener* ), considerando dependentes as mulheres que apresentaram uma resposta afirmativa ou mais^10^ .

**Figura 1:** Modelo explicativo das variáveis independentes divididas em blocos e ordem de entrada dos fatores na análise de regressão logística.

Para verificar as associações das variáveis independentes com o BP ao nascer, foi utilizada a análise de regressão logística. Os fatores associados que apresentaram p-valor inferior a 0,20 na análise bivariada por bloco foram considerados elegíveis para compor os modelos multivariados. A análise de regressão logística foi realizada seguindo a ordem de entrada dos blocos: primeiro os fatores socioeconômicos, demográficos e ambientais (bloco 1), seguidos dos fatores reprodutivos (bloco 2), fatores comportamentais (bloco 3), fatores relacionados à assistência à saúde durante o pré-natal e parto (bloco 4) e, por fim, os fatores relacionados às doenças maternas (bloco 5), conforme a Figura 1 . As variáveis anteriormente selecionadas foram submetidas à nova análise multivariada, usando-se como parâmetro para permanência no modelo final o p-valor inferior a 0,05. A pesquisa foi aprovada pelo Comitê de Ética e Pesquisa em Seres Humanos (CAAE 61055716.4.0000.5147), sendo conduzida dentro dos padrões éticos exigidos.

## RESULTADOS

A amostra foi constituída por 65 nascidos vivos pertencentes ao grupo caso e 130 ao grupo controle. Não foram incluídos no estudo um caso de BP ao nascer com diagnóstico de síndrome genética e 18 casos que não tiveram os seus respectivos controles. Nove puérperas não aceitaram participar.

Dos nascidos vivos participantes, 118 (60,5%) tinham mães que residiam em Governador Valadares e 23 (11,8%) em outros oito municípios vizinhos que também foram atingidos pela lama. Os demais 54 nascidos vivos (27,7%) tinham mães que residiam em municípios da região que não foram afetados pelo desastre.

Houve predomínio do consumo de água mineral durante a gestação (38,5%), mas 23,0% das puérperas consumiram água proveniente de poço, mina ou cisterna enquanto grávidas. As demais consumiram a água fornecida pelo serviço de abastecimento de água do município de Governador Valadares (14,9%), dos outros municípios atingidos pela lama (7,7%) e de municípios não afetados (15,9%). A única variável, dentre os fatores socioeconômicos, demográficos e ambientais, que apresentou associação significativa com o BP ao nascer na análise bivariada foi a “água consumida durante a gestação” (p = 0,008), conforme observado na Tabela 1 .

**Tabela 1:** Número de casos e controles, razão de chances, intervalo de confiança de 95% e valor de p dos fatores socioeconômicos, demográficos e ambientais (bloco1).

Variáveis	Casos (n = 65)		Controles (n = 130)		RC	IC95%	p
	
	n	%	n	%			
Escolaridade materna							
Ensino médio incompleto ou menos	32	49,2	63	48,5	Ref	-	
Ensino médio completo ou mais	33	50,8	67	51,5	0,970	0,535–1,759	0,919
Idade materna							
< 20 anos ou ≥ 35 anos	17	26,2	40	30,8	Ref	-	
20 a 34 anos	48	73,8	90	69,2	1,255	0,644–2,445	0,504
Raça materna							
Branca	11	16,9	18	13,8	Ref	-	
Negra ou parda	54	83,1	112	86,2	0,789	0,348–1,787	0,569
Situação conjugal materna							
Com companheiro	49	75,4	101	77,7	Ref	-	
Sem companheiro	16	24,6	29	22,3	1,137	0,565–2,288	0,718
Renda familiar mensal^b^							
≤ 2 salários mínimos	48	78,7	93	75,0	Ref	-	
> 2 salários mínimos	13	21,3	31	25,0	0,813	0,389–1,695	0,580
Ocupação materna							
Sem ocupação remunerada	42	64,6	84	64,6	Ref	-	
Com ocupação remunerada	23	35,4	46	35,4	1,000	0,537–1,864	1,000
Escolaridade paterna^b^							
Ensino médio incompleto ou menos	32	55,2	69	59,0	Ref	-	
Ensino médio completo ou mais	26	44,8	48	41,0	1,168	0,619–2,204	0,632
Idade paterna^b^							
< 20 anos ou ≥ 35 anos	15	24,2	37	28,7	Ref	-	
20 a 34 anos	47	75,8	92	71,3	1,260	0,629–2,526	0,514
Raça paterna							
Branca	18	27,7	32	24,6	Ref	-	
Negra, parda ou amarela	47	72,3	98	75,4	0,853	0,435–1,673	0,643
Saneamento básico							
Não	10	15,4	12	9,2	Ref	-	
Sim	55	84,6	118	90,8	0,559	0,228–1,373	0,200
Local de residência							
Urbano	50	76,9	105	80,8	Ref	-	
Rural	15	23,1	25	19,2	1,260	0,611–2,597	0,531
Água consumida durante a gestação							
Mineral, de mina, poço, cisterna ou SAA dos municípios não atingidos	43	66,2	108	83,1	Ref	-	0,008^a^
SAA dos municípios atingidos	22	33,8	22	16,9	2,512	1,262–5,00	

SAA: serviço de abastecimento de água; RC: razão de chances; IC95%: intervalo de confiança de 95%; p-valor: nível de significância estatística; Ref: categoria de referência

^a^p < 0,20.

^b^Algumas puérperas não souberam ou não aceitaram informar a renda mensal da família (n = 10), escolaridade paterna (n = 20) e idade paterna (n = 4), sendo esses dados considerados como ausentes para a análise.

No que diz respeito aos fatores reprodutivos, a variável “filho com BP ao nascer prévio” (p = 0,065) apresentou p < 0,20, assim como as variáveis “uso de cigarro” (p = 0,036) e “vítima de violência contra a gestante” (p = 0,140), referentes aos fatores comportamentais ( Tabela 2 ).

**Tabela 2:** Número de casos e controles, razão de chances, intervalo de confiança de 95% e valor de p dos fatores reprodutivos (bloco 2) e comportamentais (bloco 3).

Variáveis	Casos (n = 65)		Controles (n = 130)		RC	IC95%	p
	
	n	%	n	%			
Bloco 2							

Intervalo interpartal							
≤ 2 anos	5	7,7	15	11,5	Ref	-	0,302
> 2 anos	21	32,3	52	40,0	1,212	0,391–3,758	0,740
Primeiro filho	39	60,0	63	48,5	1,857	0,626–5,513	0,265
Aborto prévio							
Não	54	83,1	110	84,6	Ref	-	
Sim	11	16,9	20	15,4	1,120	0,501–2,505	0,782
Natimorto prévio							
Não	64	98,5	128	98,5	Ref	-	
Sim	1	1,5	2	1,5	1,000	0,089–11,236	1,000
Filho morreu antes de 1 ano							
Não	64	98,5	126	96,9	Ref	-	
Sim	1	1,5	4	3,1	0,492	0,54–4,495	0,666
Filho prematuro prévio							
Filho anterior não prematuro	25	38,5	60	46,2	Ref	-	0,218
Filho anterior prematuro	1	1,5	7	5,4	0,343	0,040–2,933	0,328
Primeiro filho	39	60,0	63	48,5	1,486	0,804–2,746	0,206
Filho com baixo peso ao nascer prévio							
Filho anterior sem baixo peso	21	32,3	63	48,5	Ref	-	0,063*
Filho anterior com baixo peso	5	7,7	4	3,1	3,750	0,921–15,275	0,065*
Primeiro filho	39	60,0	63	48,5	1,857	0,984–3,505	0,056*

Bloco 3							

Dependência de álcool							
Não	58	89,2	119	91,5	Ref	-	
Sim	7	10,8	11	8,5	1,306	0,481–3,543	0,600
Uso de cigarro							
Não	55	84,6	122	93,8	Ref	-	
Sim	10	15,4	8	6,2	2,773	1,038–7,408	0,036*
Uso de drogas							
Não	64	98,5	129	99,2	Ref	-	
Sim	1	1,5	1	0,8	2,016	0,124–32,750	1,000
Vítima de violência contra a gestante							
Não	57	87,7	122	93,8	Ref	-	
Sim	8	12,3	8	6,2	2,140	0,765–5,990	0,140*
Evento estressante na gravidez							
Não	40	61,5	89	68,5	Ref	-	
Sim	25	38,5	41	31,5	1,357	0,729–2,526	0,335

RC: razão de chances; IC95%: intervalo de confiança de 95%; p-valor: nível de significância estatística; Ref: categoria de referência

* p < 0,20.

Apenas uma puérpera, pertencente ao grupo caso, não realizou o pré-natal, não comparecendo a nenhuma consulta; três realizaram apenas uma consulta e, no total, 45 puérperas realizaram menos de seis consultas. Como a amostra seria insuficiente para a análise utilizando grupos com menor número de consultas, optou-se por classificar essa variável em apenas duas categorias, conforme descrito anteriormente. Nesse bloco, referente aos fatores relacionados à assistência à saúde durante pré-natal e parto, nenhuma variável apresentou associação significativa com o BP ao nascer ( Tabela 3 ).

**Tabela 3:** Número de casos e controles, razão de chances, intervalo de confiança de 95% e valor de p dos fatores relacionados à assistência à saúde da gestante (bloco 4).

Variáveis	Casos (n = 65)		Controles (n = 130)		RC	IC95%	p
	
	n	%	n	%			
Número de consultas no pré-natal							
< 6 consultas	18	27,7	27	20,8	Ref	-	
≥ 6 consultas	47	72,3	103	79,2	0,684	0,344–1,363	0,279
Primeira consulta do pré-natal*							
≤ 16 semanas	52	81,3	105	80,8	Ref	-	
> 16 semanas	12	18,8	25	19,2	0,969	0,451–2,082	0,936
Local do pré-natal*							
Público	54	84,4	103	79,2	Ref	-	
Privado	10	15,6	27	20,8	0,706	0,318–1,567	0,391
Vacinação na gestação							
Não	4	6,2	10	7,7	Ref	-	
Sim ou imunizada	61	93,8	120	92,3	1,271	0,383–4,218	0,695
Residência com cobertura da ESF							
Não	5	7,7	7	5,4	Ref	-	
Sim	60	92,3	123	94,6	0,683	0,208–2,241	0,527
Tipo de parto							
Vaginal	40	61,5	89	68,5	Ref	-	
Cesárea	25	38,5	41	31,5	1,357	0,729–2,526	0,335

ESF: Estratégia Saúde da Família; RC: razão de chances; IC95%: intervalo de confiança de 95%; p: nível de significância estatística; Ref: categoria de referência

* Não realizou o pré-natal uma puérpera participante, considerada ausente na análise dos dados de primeira consulta de pré-natal e local do pré-natal.

Por fim, com relação às doenças maternas, 145 puérperas apresentaram alguma durante a gestação. Nesse bloco, foram selecionadas as variáveis que apresentaram p < 0,20: “infecção do trato urinário” (p = 0,089) e “sangramento vaginal autorreferido no primeiro trimestre de gestação” (p = 0,112), como apresentado na Tabela 4 .

**Tabela 4:** Número de casos e controles, razão de chances, intervalo de confiança de 95% e valor de p das doenças maternas (bloco 5).

Variáveis	Casos (n = 65)		Controles (n = 130)		RC	IC95%	p
	
	n	%	n	%			
Diabetes gestacional							
Não	63	96,9	126	96,9	Ref	-	
Sim	2	3,1	4	3,1	1,000	0,178–5,608	1,000
Hipertensão gestacional							
Não	54	83,1	114	87,7	Ref	-	
Sim	11	16,9	16	12,3	1,451	0,631–3,339	0,379
Hipertensão crônica							
Não	63	96,9	129	99,2	Ref	-	
Sim	2	3,1	1	0,8	4,095	0,364–46,022	0,258
Pré-eclâmpsia							
Não	62	95,4	128	98,5	Ref	-	
Sim	3	4,6	2	1,5	3,097	0,504–19,012	0,336
Infecção do trato urinário							
Não	37	56,9	90	69,2	Ref	-	
Sim	28	43,1	40	30,8	1,703	0,919–3,153	0,089*
Sangramento vaginal no 1º trimestre							
Não	52	80,0	115	88,5	Ref	-	
Sim	13	20,0	15	11,5	1,917	0,851–4,316	0,112*
Oligodrâmnio							
Não	62	95,4	125	96,2	Ref	-	
Sim	3	4,6	5	3,8	1,210	0,280–5,226	1,000
Anemia							
Não	49	75,4	91	70,0	Ref	-	
Sim	16	24,6	39	30,0	0,762	0,387–1,500	0,431
Sífilis							
Não	63	96,9	125	96,2	Ref	-	
Sim	2	3,1	5	3,8	0,794	0,150–4,206	1,000

RC: razão de chances; IC95%: intervalo de confiança de 95%; p: nível de significância estatística; Ref: categoria de referência

* p < 0,20.

Na análise de regressão logística foram introduzidas, primeiramente, as variáveis “água consumida durante a gestação” (bloco 1) e “filho com BP ao nascer prévio” (bloco 2), sendo ambas mantidas no modelo. Em seguida, foram introduzidas as variáveis do bloco 3, “uso de cigarro” e “vítima de violência contra a gestante”, com retirada da última por perda de significância. Como o bloco 4 não apresentou nenhuma variável com p < 0,20, foram acrescentadas as variáveis do bloco 5: “infecção do trato urinário” e “sangramento vaginal”. Essas duas últimas variáveis também foram retiradas da análise por não apresentarem associação significativa com o BP ao nascer.

A Tabela 5 apresenta o resultado do modelo final da regressão logística, que indica que apresentaram associação significativa com o BP ao nascer: primeiro filho (razão de chances [RC] = 2,033; p = 0,036), consumo pela mãe durante a gestação de cigarro (RC = 2,850; p = 0,047) e de água fornecida pelos serviços de abastecimento dos municípios atingidos pela lama (RC = 2,444; p = 0,013).

**Tabela 5:** Resultado final da regressão logística (razão de chances, intervalo de confiança de 95% e valor de p) dos fatores associados ao baixo peso ao nascer.

Blocos	Variáveis	RC	IC95%	p
Bloco 1	Água consumida durante a gestação			
	Mineral, de mina, poço, cisterna ou SAA dos municípios não atingidos	Ref	-	
	SAA dos municípios atingidos	2,444	1,203–4,965	0,013*

Bloco 2	Filho com baixo peso ao nascer prévio			
	Filho anterior sem baixo peso	Ref	-	0,052
	Filho anterior com baixo peso	3,729	0,860–16,167	0,079
	Primeiro filho	2,033	1,047–3,948	0,036*

Bloco 3	Uso de cigarro			
	Não	Ref	-	
	Sim	2,850	1,013–8,021	0,047*

SAA: serviço de abastecimento de água; RC: razão de chances; IC95%: intervalo de confiança de 95%; p-valor: nível de significância estatística; Ref: categoria de referência

* p < 0,05.

## DISCUSSÃO

Este estudo avaliou os principais fatores associados ao BP ao nascer e os resultados demonstraram que os riscos mais elevados estão associados aos primeiros filhos e aos nascidos vivos cujas mães utilizaram cigarro durante a gestação e consumiram a água fornecida pelos serviços de abastecimento dos municípios atingidos pelos rejeitos provenientes do rompimento da barragem de Fundão. Uma das possíveis explicações para o resultado referente à água consumida durante a gestação pode estar relacionada à poluição da água do rio Doce causada por uma das maiores tragédias socioambientais do país. No dia 5 de novembro de 2015, em Mariana, Minas Gerais, mais de 70 milhões de metros cúbicos de lama de rejeitos da mineração de ferro vazaram após o rompimento da barragem pertencente à Samarco, uma *joint venture* da brasileira Vale e da anglo-australiana BHP Billiton^11^ . Além de destruir vilarejos, a lama percorreu 663 km ao longo dos rios Doce, Gualaxo do Norte e Carmo, atingindo 35 municípios em Minas Gerais e quatro no Espírito Santo^11 , 12^ .

Segundo Wanderley et al.^12^ , estudos recentes têm apresentado evidências variadas sobre a presença de metais pesados no rio e estudos anteriores já mostravam a contaminação do rio por metais, decorrente do beneficiamento mineral no alto rio Doce. A presença desses metais coloca em risco a saúde das pessoas em longo prazo, com a possibilidade de um aumento considerável de doenças crônicas^12^ . Muitas questões ainda são levantadas sobre quais os níveis de contaminação e os efeitos à saúde presentes e esperados na população exposta, em especial no solo e na qualidade da água para consumo humano^11^ . A população de Governador Valadares parece temer a contaminação da água do rio Doce, que voltou a ser fornecida para consumo humano. Tal situação pode explicar o fato de quase 40% das famílias participantes deste estudo ainda utilizarem água mineral para consumo, dois a três anos após o desastre. Agravando a situação, temos os gastos financeiros com a compra de água mineral, uma vez que a população estudada apresentou predominantemente nível econômico baixo, com renda familiar mensal inferior a dois salários mínimos.

Os recém-nascidos cujas mães, durante a gravidez, consumiram água disponibilizada pelo serviço de abastecimento dos municípios afetados pela lama de rejeitos apresentaram maior risco de BP ao nascer comparado àqueles cujas mães consumiram água mineral, de poço, mina, cisterna ou fornecida pelos municípios não afetados. Bezerra^13^ avaliou a concentração de alguns metais na água disponibilizada para consumo em Governador Valadares, por meio da análise de amostras de cinco pontos de coleta, e a comparou com a legislação específica sobre potabilidade. Os resultados mostraram que os elementos químicos alumínio, selênio e antimônio estavam elevados, em todos os pontos coletados, reprovando a água quanto ao critério de potabilidade.

O Ministério Público (MP) do estado de Minas Gerais realizou perícia para a avaliação da qualidade da água distribuída pelo serviço de abastecimento de Governador Valadares aos moradores de diversos bairros da cidade. Os resultados das análises, realizadas em 2016, demonstraram que não foram detectados metais tóxicos e prejudiciais à saúde humana em concentrações superiores aos padrões de potabilidade. No entanto, o alumínio apresentou concentrações acima dos limites estabelecidos pela Portaria de Consolidação do Ministério da Saúde nº 5, de 28 de setembro de 2017. Além disso, em alguns pontos de coleta, os parâmetros coliformes totais e turbidez da água não atendiam aos critérios de potabilidade^14^ . Considerando que os rejeitos das barragens apresentam elevadas concentrações de alumínio em sua composição, é possível que esse metal tenha sido transportado ao longo do rio Doce, ocasionando alterações na composição química em diversos trechos do curso d’água, conforme a direção dos ventos, os índices pluviométricos e a vazão do rio. Além disso, foi constatado na perícia do MP que apenas o sulfato de alumínio tem sido utilizado como coagulante para tratamento da água, tendo sido interrompido o uso do polímero de acácia negra^14 , 15^ . Os sais de alumínio são muito utilizados como coagulantes no processo de tratamento da água potável; no entanto, seu uso pode aumentar a concentração desse metal nas águas tratadas^16^ . Assim, a presença das concentrações residuais de alumínio detectadas na água destinada ao abastecimento da população pode ter como origem não somente a existência desse elemento na captação, como também na utilização de coagulante à base de sais de alumínio, no processo de tratamento e nas condições de funcionamento da estação de tratamento de água^14 , 15^ .

O ambiente materno tem implicações para a saúde do feto, sendo esse exposto transplacentariamente a contaminantes presentes nos alimentos, na água e no ar^17^ . Alguns contaminantes podem prejudicar tanto a mãe quanto o feto, a exemplo do alumínio. Os seres humanos são naturalmente expostos ao alumínio por meio da água potável, alimentos, medicamentos, poeira, produtos de higiene ou utensílios de cozinha^17^ . Apesar do conhecimento sobre a toxicidade do alumínio, pouco se sabe sobre seus efeitos em humanos, sendo a maioria dos estudos realizados em modelos com animais de laboratório. Sabe-se, entretanto, que o feto, o neonato e o lactente em desenvolvimento, devido à sua imaturidade, são mais vulneráveis à toxicidade da exposição a esse metal^18 , 19^ .

Um estudo realizado para avaliar o efeito da exposição ao alumínio, administrado em água potável, em ratas durante a gestação, lactação e pós-desmame, observou que as ratas expostas a esse componente ganharam menos peso durante a gestação e consumiram menos água e alimentos no período de lactação, e seus filhotes tiveram peso corporal diminuído comparados ao grupo controle^20^ . O alumínio ingerido pela gestante também tem efeito no metabolismo de elementos essenciais, como cálcio, magnésio, manganês, cobre, zinco e ferro^19^ . Estudo realizado com ratas grávidas tratadas com alumínio mostrou que a exposição oral a esse metal durante a gravidez pode produzir mudanças significativas na distribuição tecidual de vários elementos essenciais, com possíveis consequências no metabolismo do feto^21^ .

Outra explicação para os resultados são os possíveis agrotóxicos presentes na água consumida pela população. Dados do Ministério da Saúde e do Sistema de Informação de Vigilância da Qualidade da Água para Consumo Humano mostram que diferentes agrotóxicos foram encontrados na água de um em cada quatro municípios do Brasil entre 2014 e 2017. Foram testados 27 tipos de agrotóxicos, sendo que 16 são classificados como altamente tóxicos e 11 associados a doenças como malformação fetal e câncer. Em Governador Valadares, foram detectados 27 agrotóxicos na água que abastece a população, mas nenhum com concentração superior ao permitido pela legislação brasileira. No entanto, 24 desses agrotóxicos apresentam concentração acima do limite seguro estabelecido pela União Europeia^22^ . Um deles foi a atrazina, que está presente como uma mistura na água potável, águas subterrâneas e superficiais e pode alterar o crescimento fetal. Um estudo de coorte, realizado na França, avaliou a associação entre a exposição à atrazina durante a gravidez e os efeitos adversos ao nascimento, por meio de amostras de urina, e encontrou relação com a restrição do crescimento fetal (RC = 1,5; intervalo de confiança de 95% [IC95%] = 1,0–2,2). Os autores ressaltam que a atrazina foi proibida na Europa e que o estudo demonstra a persistência desse componente no meio ambiente, alertando os países nos quais ele ainda está em uso, como o Brasil, Estados Unidos, Argentina, México e China^23^ .

Os recém-nascidos que foram os primeiros filhos de suas mães apresentaram maior risco de BP ao nascer comparados àqueles cujas mães tiveram filhos anteriores com peso adequado. Os mecanismos biológicos de como a paridade pode influenciar na incidência de BP ainda não é bem conhecido. Uma revisão sistemática e meta-análise que investigou os riscos na gravidez para as diferentes paridades encontrou uma redução significativa do peso ao nascer, de 280 gramas, em recém-nascidos de primíparas, em relação às mulheres que já realizaram de dois a quatro partos^24^ . Um estudo realizado na Indonésia detectou um risco 46% maior (RC = 1,46; p = 0,030) de ter filhos com BP ao nascer em mulheres que nunca pariram comparado àquelas que pariram uma única vez^25^ . A taxa de BP ao nascer tende a ser maior no primeiro filho que no segundo e terceiro^26^ . Isso pode ser explicado pelo fato de ocorrer na primeira gravidez a maturação das estruturas uterinas que, nas gestações subsequentes, melhoram suas condições, permitindo maior desenvolvimento placentário e melhor nutrição fetal^27^ .

O tabagismo materno durante a gestação também merece ser discutido por apresentar riscos ao recém-nascido. Uma revisão sistemática e meta-análise investigou a relação do tabagismo materno na gravidez e BP ao nascer e encontrou que as gestantes fumantes tinham duas vezes mais chances de ter um filho com BP ao nascer em comparação com as não fumantes^28^ . Zhang et al.^29^ verificaram que nascidos de mães fumantes durante toda a gestação tiveram, as três medidas antropométricas ao nascer (peso, perímetro cefálico e comprimento) reduzidas. Tendo em vista a elevada prevalência e os efeitos adversos no desenvolvimento do sistema nervoso fetal e na morbimortalidade infantil, faz-se necessário intervir com o objetivo de reduzir o tabagismo entre as gestantes. Como o período gestacional é o mais apropriado para incentivar a cessação do tabagismo, seus efeitos nocivos para a saúde da gestante e do feto devem ser reforçados nas consultas de pré-natal pela equipe de saúde de forma mais intensa e repetida^30^ .

Uma das limitações deste estudo é o tamanho da amostra. No entanto, foram convidadas a participar todas as mães de nascidos vivos que atendiam aos critérios de inclusão. A dificuldade maior foi devida aos critérios de pareamento adotados. O pareamento por sexo foi necessário pela maior vulnerabilidade biológica masculina e associação do sexo masculino com a mortalidade perinatal e neonatal^31^ . Além disso, a literatura relata maior risco de BP ao nascer no sexo feminino^25^ , o que está de acordo com o encontrado neste estudo, no qual 50,8% dos recém-nascidos eram meninas. Já o pareamento com a data de nascimento foi realizado com o objetivo de tornar a amostra o mais semelhante possível no que diz respeito à assistência perinatal, incluindo recursos humanos e qualificação profissional da equipe presente no momento.

Outra limitação é que este estudo não avaliou a qualidade da água, apenas utilizou as informações sobre o tipo de água consumida durante a gestação. Até o momento, foram poucos os artigos publicados que tenham avaliado a qualidade da água consumida pela população de Governador Valadares e municípios vizinhos^13 , 15^ , dificultando generalizar os resultados. Ademais, não há estudos pré-desastre na região, não sendo possível afirmar que ele é responsável pelo perfil de contaminação observado^12 , 32^ . Por fim, não foi avaliada a exposição das gestantes ao alumínio presente na alimentação, no ar, nos utensílios de cozinha ou nos produtos de higiene, não permitindo assegurar que esse metal se encontra apenas na água consumida.

Conclui-se que, dentre os fatores analisados, a água consumida na gestação, primiparidade e tabagismo materno apresentaram associação com o BP ao nascer na população estudada. Reforça-se a importância de estudos epidemiológicos que avaliem a qualidade da água e seus efeitos adversos na saúde, assim como a necessidade de maior controle no pré-natal das gestantes que terão o primeiro filho e maior apoio das políticas contra o tabagismo, em especial durante a gravidez. Tornam-se necessários outros estudos para avaliar e esclarecer melhor o efeito da água sobre o BP ao nascer, sendo de fundamental importância fortalecer o monitoramento periódico da água consumida pela população.
